# *S*-nitrosoglutathione reductase alleviates morphine analgesic tolerance by restricting PKCα *S*-nitrosation^☆^

**DOI:** 10.1016/j.redox.2024.103239

**Published:** 2024-06-14

**Authors:** Ling-Yan Su, Lijin Jiao, Qianjin Liu, Xinhua Qiao, Ting Xie, Zhiyu Ma, Min Xu, Mao-Sen Ye, Lu-Xiu Yang, Chang Chen, Yong-Gang Yao

**Affiliations:** a Key Laboratory of Genetic Evolution and Animal Models of the Chinese Academy of Sciences, Yunnan Key Laboratory of Animal Models and Human Disease Mechanisms, and KIZ-CUHK Joint Laboratory of Bioresources and Molecular Research in Common Diseases, Kunming Institute of Zoology, Chinese Academy of Sciences, Kunming, Yunnan, 650204, China; b Key Laboratory of Biomacromolecules (CAS), National Laboratory of Biomacromolecules, CAS Center for Excellence in Biomacromolecules, Institute of Biophysics, Chinese Academy of Sciences, Beijing, 100101, China; c College of Food Science and Technology, and Yunnan Key Laboratory of Precision Nutrition and Personalized Food Manufacturing, Yunnan Agricultural University, Kunming, Yunnan, 650201, China; d Kunming College of Life Science, University of Chinese Academy of Sciences, Kunming, Yunnan, 650204, China; e National Research Facility for Phenotypic & Genetic Analysis of Model Animals (Primate Facility), National Resource Center for Non-Human Primates, Kunming Institute of Zoology, Chinese Academy of Sciences, Kunming, Yunnan, 650107, China

**Keywords:** Analgesic tolerance, Morphine, PKCα, GSNOR, *S*-nitrosation

## Abstract

Morphine, a typical opiate, is widely used for controlling pain but can lead to various side effects with long-term use, including addiction, analgesic tolerance, and hyperalgesia. At present, however, the mechanisms underlying the development of morphine analgesic tolerance are not fully understood. This tolerance is influenced by various opioid receptor and kinase protein modifications, such as phosphorylation and ubiquitination. Here, we established a murine morphine tolerance model to investigate whether and how *S*-nitrosoglutathione reductase (GSNOR) is involved in morphine tolerance. Repeated administration of morphine resulted in the down-regulation of GSNOR, which increased excessive total protein *S*-nitrosation in the prefrontal cortex. Knockout or chemical inhibition of GSNOR promoted the development of morphine analgesic tolerance and neuron-specific overexpression of GSNOR alleviated morphine analgesic tolerance. Mechanistically, GSNOR deficiency enhanced *S*-nitrosation of cellular protein kinase alpha (PKCα) at the Cys78 and Cys132 sites, leading to inhibition of PKCα kinase activity, which ultimately promoted the development of morphine analgesic tolerance. Our study highlighted the significant role of GSNOR as a key regulator of PKCα *S*-nitrosation and its involvement in morphine analgesic tolerance, thus providing a potential therapeutic target for morphine tolerance.

## Highlights

• Chronic morphine exposure alters GSNOR expression and total protein *S*-nitrosation.
• GSNOR deficiency or chemical inhibition promotes morphine analgesic tolerance *in vivo*.
• GSNOR deficiency enhances *S*-nitrosation of PKCα and inhibits PKCα activity.
• Inhibition of PKCα by GO6976 promotes morphine analgesic tolerance in mice.

## 1 Introduction

Morphine, a natural opioid, acts directly on the central nervous system to relieve pain [1]. However, prolonged and repeated use of morphine can lead to the development of morphine tolerance, which reduces its effectiveness and clinical utility and can give rise to addictive behaviors and hyperalgesia [2,3]. Despites its significance, the exact mechanisms underlying morphine analgesic tolerance are insufficiently understood but are crucial for developing effective strategies aimed at preventing or reversing such tolerance. Notably, opioid receptor desensitization, internalization, excessive production of nitric oxide (NO), and neuroinflammation are reported to be closely related to morphine tolerance [[2], [3], [4], [5], [6], [7], [8]].

Increasing evidence has highlighted the involvement of post-translational modifications (PTMs), which affect protein function by regulating their abundance and activity, in the development of morphine analgesic tolerance [9]. As opioid receptor activity plays a central role in the development of tolerance to and dependence on morphine and other addictive substances, understanding the molecular mechanisms regulating receptor activity is of fundamental importance. Among the well-established PTMs, glycosylation (sugar-linkage) [10,11], palmitoylation (palmitoyl-linkage) [12,13], phosphorylation (phosphate-linkage) [[14], [15], [16], [17]] and ubiquitination (ubiquitin-linkage) [[18], [19], [20]], are most frequently implicated in controlling morphine analgesic tolerance via reversible PTM of opioid receptors and downstream signaling molecules. *S*-nitrosation, a reversible PTM, crucially regulates NO-related and redox signaling pathways by forming covalent interactions between NO and the thiol group of cysteine residues [21]. *S*-nitrosoglutathione reductase (GSNOR), a highly evolutionarily conserved enzyme of the denitrosylating enzymatic system, modulates *S*-nitrosation through catabolism of *S*-nitrosoglutathione (GSNO) [22]. Increased *S*-nitrosation of proteins has been reported in mice with genetic deletion of *Gsnor* [23], which is associated with various diseases, including cancer, cardiovascular disease, neurodegenerative disease, and metabolic disorders [[24], [25], [26], [27]]. We recently found that GSNOR deficiency attenuates MPTP-induced neurotoxicity and autophagy by facilitating CDK5 *S*-nitrosation in a mouse model of Parkinson's disease [28]. GSNOR facilitates antiviral innate immunity by restricting *S*-nitrosation of TANK-binding kinase 1 [29]. Increased GSNOR expression during aging can impair cognitive function and decrease *S*-nitrosation of calcium/calmodulin-dependent protein kinase II alpha [30]. Considering the crucial role of opioid receptor activity in the development of morphine tolerance and addiction, we hypothesize that GSNOR-regulated *S*-nitrosation of proteins could play a pivotal role in this process.

Using cellular and animal models (*Gsnor* knockout (KO) mice and neuron-specific GSNOR overexpression (*Gsnor* TG) mice) with morphine administration, we demonstrated the active involvement of GSNOR in morphine analgesic tolerance. We uncovered the underlying mechanism that KO or inhibition of GSNOR enhanced *S*-nitrosation of protein kinase alpha (PKCα) on Cys78 and Cys132 and reduced PKCα kinase activity, which ultimately promoted the development of morphine-induced analgesic tolerance. These novel findings highlight the involvement of GSNOR in the development of morphine tolerance via the regulation of PKCα *S*-nitrosation *in vivo* and *in vitro*, thus providing a potential therapeutic target for morphine tolerance.

## 2 Materials and methods

### 2.1 Reagents, antibodies, and cell culture

The reagents and antibodies used in this study are listed in Table S1. Morphine hydrochloride was purchased from Shenyang Pharmaceutical Co. Ltd. (State Drug Approval Document Number: H21022436; Shenyang, China). Rat pheochromocytoma PC12 cells were obtained from the Kunming Cell Bank, Kunming Institute of Zoology (China). *Gsnor* KO PC12 cells, first described in our recent study [28], were constructed using CRISPR/Cas9 [31]. We chose the PC12 cell line as a cellular model to detect the neurotoxic effects of morphine on *S*-nitrosation. PC12 cells are commonly used in neuroscience research [32] and were effectively genetically modified in our study. The cells were maintained in Dulbecco's Modified Eagle Medium (DMEM) (Gibco-BRL, 11 965–092) supplemented with 10 % fetal bovine serum (FBS, Gibco-BRL, 10 099–141) and 1 × penicillin/streptomycin (Gibco, 15 140 122) at 37 °C in a humidified atmosphere incubator with 5 % CO_2_ and 95 % humidity. Drugs were added directly to the culture medium for treatment. All cellular experiments were performed at least three times, unless otherwise specified.

### 2.2 Plasmids and transfection

Expression vectors for PKCα (PKCα-WT) and site-directed mutants of PKCα (p.C67S (PKCα-p.C67S), p. C78S (PKCα-p.C78S), p. C86S (PKCα-p.C86S), and p. C132S (PKCα-p.C132S)) were purchased from the Miaoling Plasmid Sharing Platform (MLPSP, Wuhan, China). All constructs were confirmed by Sanger DNA sequencing. Transient transfection of each vector was performed using Lipofectamine™ 3000 (Invitrogen, L3000015) according to the manufacturer's protocols.

### 2.3 Biotin-switch assay and quantitative *S*-nitrosation proteomic analysis

The *S*-nitrosation modification of proteins was performed in the dark, as described previously [33,34]. Briefly, cells and brain tissue samples were homogenized in HEN buffer (250 mM HEPES-NaOH (pH 7.7), 1 mM EDTA, and 0.1 mM neocuproine) with 1 % (v/v) NP-40. The free cysteine thiols were blocked with blocking buffer (2.5 % sodium dodecyl sulphate (SDS), 20 mM methyl methanethiosulfonate (MMTS; Sigma-Aldrich, 208 795-1G) in HEN buffer) at 50 °C for 30 min with frequent vortexing. Excessive MMTS was removed by ice-cold acetone precipitation for 30 min, followed by centrifugation at 2000×*g* for 10 min at 4 °C. Precipitation was repeated three times to remove residual MMTS. The protein pellet was resuspended in HEN buffer (250 mM HEPES-NaOH (pH 7.7), 1 mM EDTA, 0.1 mM neocuproine, and 1 % SDS) with 0.4 mM sulfhydryl-specific biotinylating reagent *N*-(6-(biotinamido) hexyl)-30-(20-pyridyldithio) propionamide (biotin-HPDP) and 10 mM ascorbate, then incubated for 2 h at room temperature. Excessive biotin-HPDP was removed by ice-cold acetone precipitation for 30 min, followed by centrifugation at 2000×*g* for 10 min at 4 °C. The protein pellet was resuspended in HEN buffer, with streptavidin-agarose beads and three volumes of neutralization buffer (20 mM HEPES-NaOH (pH 7.7), 100 mM NaCl, 1 mM EDTA) added for overnight incubation at 4 °C. The beads were then washed five times with neutralization buffer with 0.6 M NaCl and eluted using 30 μL of elution buffer (20 mM HEPES-NaOH (pH 7.7), 100 mM NaCl, 1 mM EDTA, 100 mM β-mercaptoethanol). Biotinylated proteins were heated at 100 °C for 5 min in reducing sodium dodecyl sulphate-polyacrylamide gel electrophoresis (SDS-PAGE) loading buffer and analyzed by SDS-PAGE. Finally, the biotinylated proteins were detected by immunoblotting using appropriate antibodies. The Pierce™ *S*-Nitrosylation Western Blot Kit (ThermoFisher, 90 105) was used to detect the total *S-*nitrosation of protein according to the manufacturer's protocols. For the positive and negative controls, lysates of prefrontal cortex tissues or PC12 cells were incubated with GSNO (500 μM) or GSH (500 μM) for 30 min, then subjected to a biotin-switch assay in the presence or absence of ascorbate.

Quantitative *S*-nitrosation (SNO) proteomic analysis and liquid chromatography tandem-mass spectrometry (LC-MS/MS) determination of the *S*-nitrosation sites were performed after total SNO modification according to our previous study [35,36]. Briefly, proteins from 5 wild-type (WT) and 5 *Gsnor* KO mice (treated with or without morphine; each group, *n* = 5) were mixed and treated with MMTS to block free cysteine thiols, and *S*-nitrosation sites were reduced with ascorbate and labeled with iodoacetyl tandem mass tag (iodoTMT) using an iodoTMT labeling kit (Thermo Fisher Scientific). Then, the proteins from the above four groups of samples were then mixed and digested by sequencing-grade modified trypsin (0.3 g of trypsin to digest 10 g of protein). After incubation at 37 °C overnight, trypsin digestion was terminated by further incubation at 20 °C for 30 min. The protein digests were dried in a centrifugal vacuum concentrator, and 20 μL of triethylammonium bicarbonate (TEAB) buffer was used to suspend the freeze-dried peptide. The iodoTMT reagent-labeled peptides were enriched with *anti*-TMT™ antibody resin. After suspension of the mixed samples with spin tip C18 desalination, the peptides were analyzed on a Q-Exactive high-resolution mass spectrometry (Thermo Scientific) equipped with the Easy *n*-LC 1000 HPLC system (Thermo Scientific). The ratio of protein *S*-nitrosation between groups was analyzed, and protein *S*-nitrosation ratios up-regulated (>1.2) or down-regulated (<0.8) were defined as significantly different between two groups.

### 2.4 Western blotting

Western blot assays for the target proteins were performed as described in our previous studies [7,37,38]. In brief, cell lysates of different mouse prefrontal cortex tissues and cultured PC12 and SH-SY5Y cells were prepared using protein lysis buffer (Beyotime Institute of Biotechnology, P0013). Protein concentration was determined using a BCA Protein Assay Kit (Beyotime Institute of Biotechnology, P0012). In total, 25 μg of protein was separated by 12 % or 15 % SDS-PAGE and transferred to a polyvinylidene difluoride membrane (Bio-Rad, L1620177 Rev D). The membrane was soaked with 5 % (w:v) skim milk for 2 h at room temperature, then incubated with primary antibodies against GSNOR (1:1000), PKCα (1:1000), tubulin (1:10 000), and β-actin (ACTB; 1:10 000) overnight at 4 °C. The membranes were washed three times (5 min each time) with Tris-buffered saline (Cell Signaling Technology, 9997) with Tween 20 (0.1 %; Sigma, P1379), followed by incubation with peroxidase-conjugated anti-mouse or anti-rabbit IgG (1:10 000) for 1 h at room temperature. The epitope was visualized using an ECL Western Blot Detection Kit (Millipore, WBKLS0500). ImageJ (National Institutes of Health, Bethesda, Maryland, USA) was used to evaluate densitometry. Western blotting for tubulin or ACTB was used as a loading control to measure the densitometry of GSNOR and PKCα.

### 2.5 PKCα activity assay

The PKCα kinase assay was conducted using a PKC Kinase Activity Assay Kit (Abcam, ab139437) according to the manufacturer's protocols, with some modifications. In brief, cell samples were homogenized in ice-cold lysis buffer (Beyotime Institute of Biotechnology, P0013) for 1 h, followed by centrifugation at 12 000×*g* for 10 min at 4 °C. Protein concentration was quantified using a BCA Protein Assay Kit (Beyotime Institute of Biotechnology, P0012). To obtain the specific PKCα isoform, immunoprecipitation (IP) was performed before the activity assay. Anti-Flag antibodies (for exogenously expressed PKCα kinase activity assay in cultured cells) or *anti*-PKCα antibodies (for endogenous PKCα kinase activity assay in mouse tissues) were incubated with protein G-agarose beads (Life Technologies, 15 920 010) to form a complex for 1 h at room temperature, followed by the addition of 1 mg of protein and overnight incubation at 4 °C. The beads were collected and washed five times with phosphate-buffered saline (PBS). After sample preparation, PKCα kinase activity was detected according to the manufacturer's protocols.

### 2.6 Animals

Male C57BL/6 mice were obtained from the Animal Core Facility of the Experimental Animal Center of the Kunming Institute of Zoology (KIZ, China). The *Gsnor*^*−/−*^ mice (*Gsnor* KO) on a C57BL/6 J background have been described in our previous studies [29,30] and were compared with age-matched littermates. Thy1-EGFP (TG) mice overexpressing GSNOR exclusively in neurons (*Gsnor* TG mice) were obtained from the Jackson Laboratory, as described in our previous study [30]. Mice were genotyped using polymerase chain reaction (PCR) amplification (Table S2). All mice were bred in a specific pathogen-free animal house at the Experimental Animal Center of KIZ, with free access to water and food under 22 ± 2 °C, 50 % humidity, and 12 h light/dark cycle conditions. For all behavioral tests, 8-week-old male WT, *Gsnor* KO, and *Gsnor* TG littermates were used.

### 2.7 Morphine analgesia and tolerance

Analgesia was assessed using radiant heat tail-flick latency (Tail-Flick Unit 37 360, UGO Baseline, Comerio, Italy) and a hot platform (BIO–CHP, Bioseb, France), as described in our previous studies [7,38,39]. For the tail-flick test, mice were placed in Plexiglas cages (9 × 6 × 3 cm) on a modified Hargreaves Device (Panlab HARVARD, MA, USA). Mice were habituated to the device for 2 min before each test session. A halogen lamp was focused on the tail and withdrawal reflex time was determined using a photocell. Tail-flick latency was measured at IR30 (decimal selector of heat intensity) and IR50 every two days from Day 0 (baseline) to Day 7. To avoid damaging the tail, 20 s was used as the cutoff time. Baseline responses were determined for each mouse before drug injection. For morphine hot-plate tolerance, repeated morphine injections (10 mg/kg subcutaneously) were given daily for 7 consecutive days. The hot-plate test was performed on a platform heated to 47.5 °C, 50 °C, and 52.5 °C, with a cutoff of 30 s and latency to paw lick or jump recorded. Baseline responses were determined for each animal before treatment. The analgesic response to morphine was assessed using the tail-flick and hot plate tests 1 h after morphine injection (10 mg/kg). The antinociceptive response was calculated as a percentage of maximal possible effect (MPE), where MPE%=(test latency–baseline latency)/(cutoff latency–baseline latency) × 100 [40,41].

### 2.8 Von Frey test for mechanical allodynia

For mechanical allodynia assessment, Von Frey testing [42] was applied with ascending forces expressed in grams (0.001–300 g; Electronic Von Frey Anesthesiometer; IITC). Each filament was applied five times in a row against the lateral area of the paw. Hindpaw withdrawal or licking induced by the filament was defined as a positive allodynia response. A positive response in three out of five repetitive stimuli was defined as the allodynia threshold. Mice were habituated to the Von Frey apparatus for 30 min before the test. For tolerance studies, mice were injected subcutaneously with 10 mg/kg morphine for 7 consecutive days, with the Von Frey test assessed 1 h after injection every two days from Day 0 (baseline) to Day 7.

For all behavioral experiments, data acquisition and analyses were performed using a double-blind, controlled design. All animal experimental procedures and protocols were approved by the Institutional Review Board of KIZ, Chinese Academy of Sciences (CAS) (approval no: SMKX-20190310-39).

### 2.9 Statistical analysis

Comparisons of relative protein levels were performed using unpaired two-tailed Student's *t*-test. Differences in animal analgesic tolerance were determined by analysis of variance (ANOVA), followed by the Least Significant Difference (LSD) test for *post-hoc* comparisons, as described in our previous study [7]. Data were represented as mean ± standard error of the mean (SEM) or mean ± standard deviation (SD). A *P-*value less than 0.05 was considered statistically significant.

## 3 Results

### 3.1 Chronic morphine exposure decreased GSNOR expression and increased total protein *S*-nitrosation

**Fig. 1:**
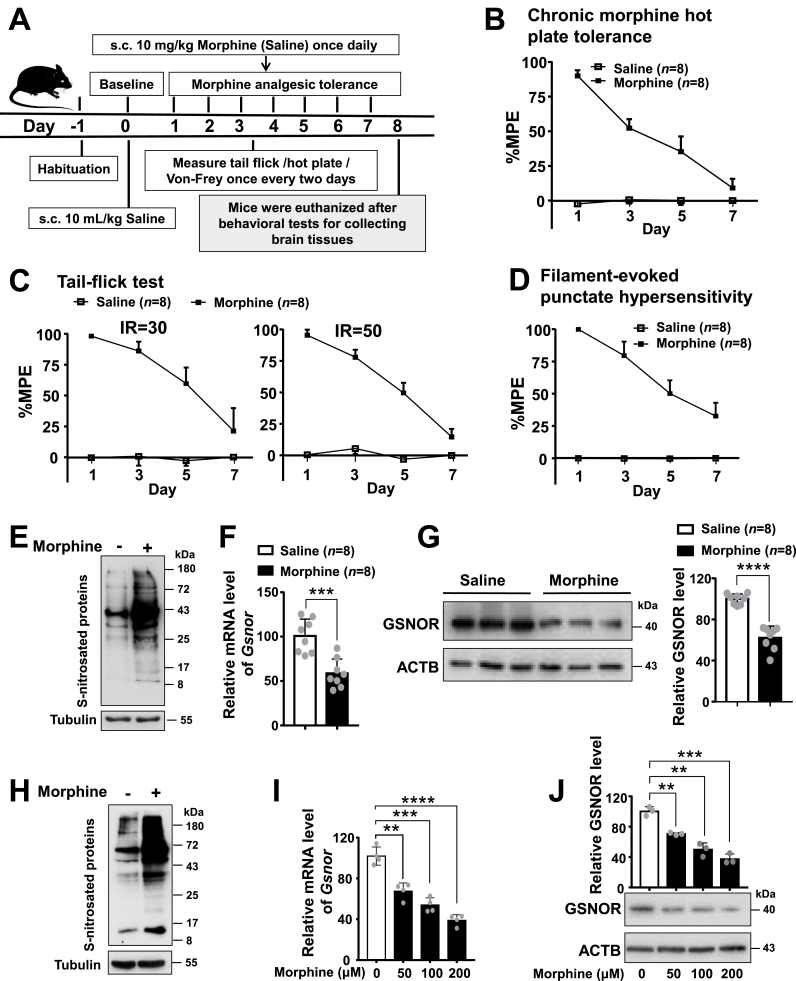
Chronic morphine exposure increased total protein *S*-nitrosation levels and decreased GSNOR expression levels in prefrontal cortex tissues of wild-type (WT) mice and in PC12 cells (**A**) A schematic profile illustrating the experimental design of hot plate, tail-flick, Von Frey tests and tissues collection for WT with chronic morphine treatment. (**B-D**) Chronic morphine administration (10 mg/kg once daily) induced analgesic tolerance in WT mice, as mirrored by the gradual decrease in percentage of maximal possible effect (MPE%) in the hot plate (**B**), tail-flick (laser density of IR30 and IR50) (**C**), and Von Frey tests (**D**) (*n* = 8 animals per group). Antinociceptive response was calculated as MPE%=(test latency−baseline latency)/(cutoff latency−baseline latency) × 100. Bars refer to mean ± SEM. (***E*-G**) Chronic morphine exposure led to increased total protein *S*-nitrosation (**E**) and decreased *Gsnor* mRNA (**F**) and protein expression (**G**) in prefrontal cortex tissues of WT mice (*n* = 8 animals per group, tissues were collected after tests in **B-D**). (**H-J**) Morphine treatment caused alterations of total protein *S*-nitrosation (**H**) and decreased *Gsnor* mRNA (**I**) and protein expression levels (**J**) in PC12 cells. Cells were grown in DMEM supplemented with 10 % FBS and 1 × penicillin/streptomycin to 80 % of confluence, then were treated with or without morphine treatment (200 μM) for 24 h before harvest for analyzing total protein *S*-nitrosation (**H**). Similarly, PC12 cells were cultured and treated with various concentrations of morphine (0, 50, 100, and 200 μM) for 24 h before harvest for quantifying *Gsnor* mRNA (**I**) and protein (**J**) levels. Results are representative of three independent experiments with similar results. All results are presented as mean ± SD. Group differences were analyzed by unpaired two-tailed *t*-test. **, *P* < 0.01; ***, *P* < 0.001; ****, *P* < 0.0001.

We established a chronic morphine-tolerant mouse model following our previous studies [7,38,39], and observed antinociceptive tolerance in response to morphine based on behavioral tests (Fig. 1). Consistent with our prior studies [7,38,39], administration of morphine (10 mg/kg, subcutaneous (s.c.)) for 7 consecutive days (Fig. 1A) well established antinociceptive tolerance, as revealed by standard hot-plate tests (Fig. 1B), tail-flick tests (Fig. 1C), and Von Frey tests (Fig. 1D). We initially investigated the potential association between *S*-nitrosation of protein and morphine analgesic tolerance by measuring the total protein *S*-nitrosation (SNO-protein) level in response to chronic morphine treatment. The prefrontal cortex tissues were chosen for analyses given the relatively ample samples of this brain region and the key role of the cortex (including anterior cingulate cortex, insular cortex, primary and secondary somatosensory cortices, and prefrontal cortex) in controlling pain [43]. Results showed that chronic morphine exposure increased the SNO-protein level in the prefrontal cortex tissues of mice (Fig. 1E), who finished the related behavioral tests showing antinociceptive tolerance induced by morphine (Fig. 1B–D). Concordantly, we observed a significant decrease of the relative levels of mRNA (Fig. 1F) and protein (Fig. 1G) of GSNOR in the cortex of mice treated with morphine compared to those without. We further validated the effect of morphine treatment as observed in mouse cortex tissues by using cellular models. Morphine treatment remarkably increased the SNO-protein level in PC12 cells (Fig. 1H) and SH-SY5Y cells (Fig. S1). We also observed a significant decrease of the mRNA (Fig. 1I) and protein levels of GSNOR (Fig. 1J) in a dose-dependent manner in PC12 cells with morphine treatment.

Considering the fact that previous studies showed morphine induces apoptosis in cultured cancer cell lines and other types of cells at micromolar concentrations after treatment for hours to a few days [[44], [45], [46]] and neuronal cell death and apoptosis are involved in morphine analgesic tolerance [47,48] we detected the cell viability after morphine treatment by using Cell Counting Kit-8 (CCK-8) assay. We found no apparent difference of cell viability in PC12 (Fig. S2A) and SH-SY5Y (Fig. S2B) cells with and without morphine treatment for 24 h, suggesting that the cells are tolerant to short-term morphine treatment with the indicated concentration in our experiments. However, we found that morphine increased the NO level (Fig. S3A) and the GSSG/GSH ratio (Fig. S3B), which is an indicative of oxidative stress [49], in cultured PC12 cells with morphine treatment for 24 h in a dose-dependent manner. Consistent with previous reports [50], we found that chronic morphine exposure to mice for 7 consecutive days induced the glial fibrillary acidic protein (GFAP) expression in astrocytes in the prefrontal cortex tissues (Fig. S4), which is indicative of inflammation and neural stress induced by morphine treatment.

Taken together, all these findings indicate that morphine can increase SNO-protein levels but decrease GSNOR expression in both mouse models and cellular models, thus suggesting a potentially active role of GSNOR in the process of morphine analgesic tolerance.

### 3.2 GSNOR was crucial for morphine analgesic tolerance

**Fig. 2:**
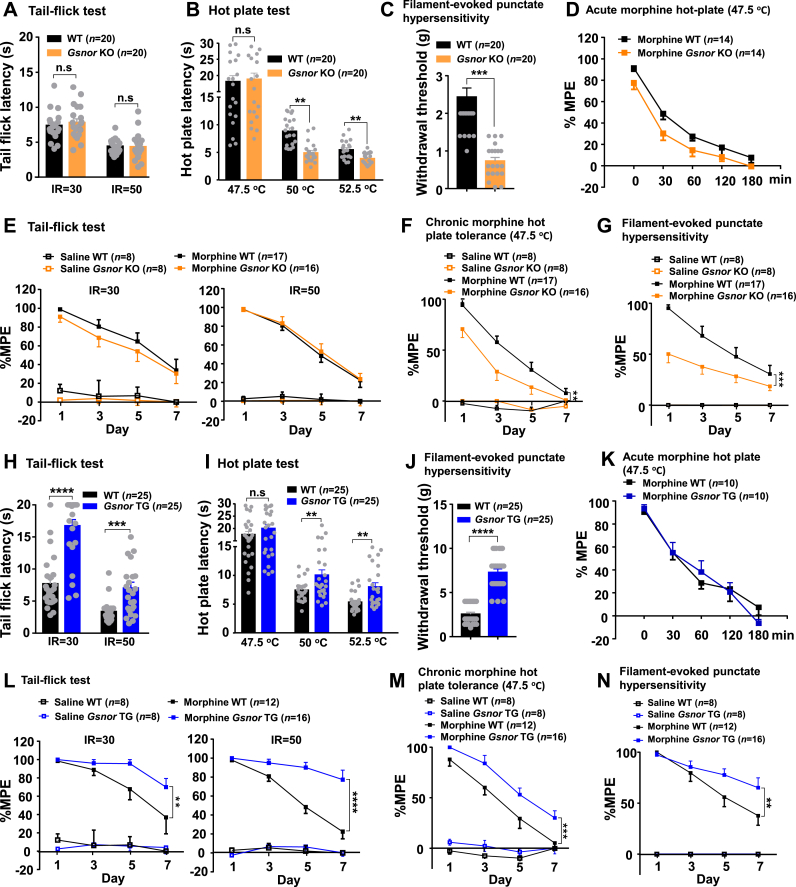
GSNOR affected morphine-induced analgesic tolerance **(A**–**D)***Gsnor* KO mice exhibited normal nociceptive thresholds in the tail-flick test (**A**) (*n* = 20 animals per group) and hot plate test at 47.5 °C (**B**), but a lower nociceptive threshold in the hot plate tests at 50 °C and 52.5 °C (**B**) and Von Frey test **(C)** compared to their WT littermates (*n* = 20 animals per group). Analgesic responses to morphine (10 mg/kg, subcutaneously) in the hot plate test at 47.5 °C were approximately the same in WT and *Gsnor* KO mice, and duration time of analgesia was similar (**D**) (*n* = 14 animals per group). **(*E***–**G)** WT mice with chronic morphine administration (10 mg/kg once daily) exhibited analgesic tolerance, as mirrored by the gradual decrease in MPE% in the tail-flick (**E**), hot plate at 47.5 °C (**F**), and Von Frey tests (**G**). *Gsnor* KO mice showed similar analgesic tolerance in the tail-flick test (**E**) and increased analgesic tolerance in the hot plate at 47.5 °C (**F**) and Von Frey tests (**G**) (*n* = 8–17 animals per group). **(H–K)** Mice overexpressing GSNOR in neurons (*Gsnor* TG mice) exhibited a higher nociceptive threshold in the tail-flick test (**H**), hot plate tests at 50 °C and 52.5 °C (**I**), and Von Frey test (**J**) compared to WT littermates (*n* = 25 animals per group). Analgesic responses to morphine (10 mg/kg, subcutaneously) in the hot plate test at 47.5 °C were approximately the same in WT and *Gsnor* TG mice, and duration time of analgesia was similar (**K**) (*n* = 10 animals per group). **(L**–**N)***Gsnor* TG mice showed delayed analgesic tolerance to morphine in the tail-flick test (**L**) and increased analgesic tolerance in the hot plate at 47.5 °C (**M**) and Von Frey tests (**N**). All results are presented as mean ± SEM (*n* = 8–12 animals per group). Group differences were analyzed by two-way ANOVA with the Tukey's *post-hoc* test (**A-C, H-J**) or two-way repeated-measures ANOVA (**D-G, K–N**). **, *P* < 0.01; ***, *P* < 0.001; ****, *P* < 0.0001; n. s, not significant.

Next, we investigated whether the morphine-induced analgesic effects could be affected by GSNOR using *Gsnor* KO and *Gsnor* TG mice. The *Gsnor* KO mice showed normal nociceptive thresholds in the tail-flick test (laser density of IR30 and IR50) (Fig. 2A) and hot-plate test at 47.5 °C (Fig. 2B) but presented a significantly lower nociceptive threshold in the hot-plate test at 50 °C and 52.5 °C (Fig. 2B) and Von Frey test (Fig. 2C) compared to their wild-type (WT) littermates. The duration of morphine activity was not affected by *Gsnor* deficiency, as the analgesic effects persisted for similar amounts of time in both the *Gsnor* KO and WT mice (Fig. 2D), although the *Gsnor* KO mice showed an overall lower value of hot-plate latency relative to the WT mice (Fig. 2D). Furthermore, both the *Gsnor* KO and WT mice showed a declined tail-flick latency (laser density of IR30 and IR50) (Fig. 2E) and developed hyperalgesia (Fig. 2F–G) after repeated morphine administration over 7 days. Of note, the *Gsnor* KO mice exhibited a relatively lower value of latency in response to chronic morphine administration (10 mg/kg) in both the hot-plate test at 47.5 °C (Fig. 2F) and Von Frey test (Fig. 2G). Conversely, the *Gsnor* TG mice demonstrated a significantly higher nociceptive threshold in the tail-flick test (Fig. 2H), hot-plate tests at 50 °C and 52.5 °C (Fig. 2I), and Von Frey test (Fig. 2J) compared to their WT littermates. The analgesic responses to acute morphine treatment (10 mg/kg, subcutaneously) were approximately the same between the WT and *Gsnor* TG mice in the hot-plate test at 47.5 °C (as indicated by the similar duration of analgesia; Fig. 2K), suggesting that GSNOR overexpression did not affect morphine metabolism in mice. The *Gsnor* TG mice showed significantly delayed analgesic tolerance to chronic morphine treatment in the tail-flick test (laser density of IR30 and IR50, Fig. 2L), hot plate test at 47.5 °C (Fig. 2M), and Von Frey test (Fig. 2N). Compared to the WT littermates, the *Gsnor* KO mice exhibited an overall lower hot-plate latency (Fig. 2F), while the *Gsnor* TG mice exhibited a higher hot-plate latency (Fig. 2M). The exact reason underlying this pattern remains to be determined.

We used a specific and potent GSNOR inhibitor (N6022) [51] to mimic the effect of *Gsnor* KO during the development of morphine analgesic tolerance. Consistent with the observations in *Gsnor* KO mice, N6022 treatment had no effect on mice with and without morphine treatment in any of the comparative analyses (saline group versus N6022 group; morphine group versus morphine and N6022 group) in the tail-flick test (Fig. S5A). However, mice injected with N6022 were more susceptible to the development of morphine tolerance than the control mice in the hot plate test (Fig. S5B) and Von Frey test (Fig. S5C). Collectively, these results suggest that GSNOR is actively involved in the development of analgesic responses and tolerance to repeated morphine administration.

### 3.3 Morphine and GSNOR regulate PKCα *S*-nitrosation and PKCα kinase activity

**Fig. 3:**
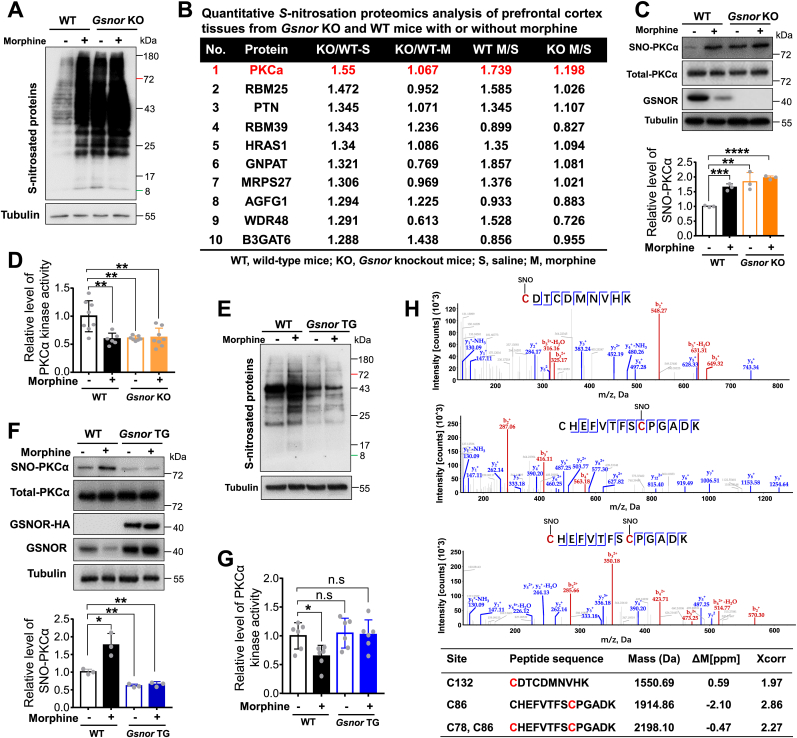
GSNOR affected morphine-induced *S*-nitrosation of PKCα and PKCα kinase activity **(A)** Chronic morphine exposure or *Gsnor* KO increased total protein *S*-nitrosation in prefrontal cortex tissues of mice. Wild-type (WT) and *Gsnor* KO mice received chronic morphine (10 mg/kg) treatment or saline for 7 consecutive days before euthanasia for collecting prefrontal cortex tissues. (**B)** Top 10 proteins captured by quantitative *S*-nitrosation proteomics analysis of prefrontal cortex tissues from *Gsnor* KO and WT mice with or without morphine treatment. Prefrontal cortex tissue proteins from 5 wild-type (WT) and 5 *Gsnor* KO mice were mixed and used for the assay. (**C)** Levels of *S*-nitrosated PKCα and total PKCα in prefrontal cortex tissues of WT and *Gsnor* KO mice with or without morphine treatment (*n* = 3 mice per group). Shown data are Western blot images for SNO-PKCα and related proteins (*upper*) and quantification of SNO-PKCα level relative to Tubulin (*below*). (**D)** Chronic morphine administration and GSNOR deficiency inhibited PKCα kinase activity in prefrontal cortex of mice (*n* = 6–8 mice per group). **(*E***–**F)** Overexpression of GSNOR in neurons (*Gsnor* TG) decreased total protein *S*-nitrosation (**E**) and *S*-nitrosated PKCα levels (**F**) in the prefrontal cortex of mice with or without morphine treatment (*n* = 3 mice per group). Shown data in (**F**) are Western blot images for SNO-PKCα and related proteins in WT and *Gsnor* TG mice with or without morphine treatment (*upper*), and quantification of SNO-PKCα level relative to Tubulin (*below*). **(G)** Chronic morphine administration had no effect on PKCα kinase activity in the prefrontal cortex tissues of *Gsnor* TG mice but decreased PKCα kinase activity of WT mice (*n* = 6 mice per group). **(H)** Tandem mass spectrum of peptides showing *S*-nitrosation of Cys132, Cys86, and Cys78 in GSNO-treated prefrontal cortex tissue lysates. Summary of biotin-*M*-containing peptides is shown, including calculated monoisotopic mass (mass), accuracy of mass measurements in parts per million (ppm), and cross correlation score (Xcorr). Cs marked in red indicate biotinylation sites. All results are presented as mean ± SD. Group differences in (**C**, **D**, **F** and **G**) were analyzed by two-way ANOVA with Tukey's *post-hoc* test. *, *P* < 0.05; **, *P* < 0.01; ***, *P* < 0.001; ****, *P* < 0.0001; n. s, not significant. (For interpretation of the references to colour in this figure legend, the reader is referred to the Web version of this article.)

To identify the potential target mediating the regulatory effects of GSNOR during the development of morphine analgesic tolerance, we detected the SNO-protein level in the prefrontal cortices of WT and *Gsnor* KO mice with and without morphine treatment. We used GSNO as a positive control and glutathione (GSH) as a negative control [28,29]. Prefrontal cortex tissue lysates from WT mice were incubated with GSNO (500 μM) or GSH (500 μM) for 30 min, then subjected to *S-*nitrosation assay. Total *S-*nitrosation level was increased in GSNO treatment group and decreased in GSH treatment group compared with the un-treatment group (Fig. S6A), suggesting that the experimental system was feasible. Chronic morphine administration in the WT mice resulted in a significant increase in the SNO-protein level in the prefrontal cortex compared to that in the control mice (Fig. 3A). A similar pattern of morphine-induced SNO-protein was observed in the prefrontal cortices of *Gsnor* KO mice with and without morphine injection. The basal SNO-protein level was much higher in the *Gsnor* KO mice than in the WT mice, consistent with the effects of GSNOR deficiency (Fig. 3A) and GSNOR inhibition by N6022 in WT mice (Fig. S6B). These results indicate that morphine increases the SNO-protein level, with further enhancement by GSNOR loss or inhibition.

We next performed liquid chromatography-tandem mass spectrometry (LC-MS/MS)-based quantitative proteomics analysis of SNO*-*protein by isobaric tandem mass tag (TMT) labeling of prefrontal cortex proteins in mice with or without morphine to identify potential targets with the highest *S*-nitrosation. Within the prefrontal cortex tissues of *Gsnor* KO and WT mice, PKCα emerged as the primary protein among the top 10 proteins exhibiting increased SNO-protein levels (Fig. 3B). PKCα was also identified in comparisons involving both WT mice with or without morphine and KO mice with or without morphine. This finding is mostly consistent with previous studies reporting direct participation of PKCα in morphine analgesic tolerance [1,52]. It has also been reported that PKCα can be *S*-glutathionylated and *S*-nitrosylated in other systems [[53], [54], [55], [56]]. Therefore, we speculate that the *S*-nitrosation of PKCα may be a novel regulatory mechanism underlying its role in morphine analgesic tolerance.

We first validated the specificity of the PKCα *S*-nitrosation signal in the prefrontal cortex tissues of WT mice using the biotin-switch assay. PKCα was readily *S*-nitrosated in tissues after GSNO treatment, while no PKCα *S*-nitrosation signal was detected in tissues treated with GSH (Fig. S6C), indicating that our detection system for PKCα *S*-nitrosation was robust. Next, we performed the biotin-switch assay to detect the potential effects of GSNOR on the *S*-nitrosation of PKCα. In WT mice, the level of PKCα *S-*nitrosation was significantly increased by morphine (Fig. 3C). We observed a high level of PKCα *S*-nitrosation in the prefrontal cortex of *Gsnor* KO mice, regardless of morphine treatment (Fig. 3C). To determine whether GSNOR regulates morphine analgesic tolerance via PKCα activity, we performed an *in vitro* kinase assay to quantify PKCα activity. Results showed that morphine treatment and GSNOR deficiency significantly decreased PKCα kinase activity (Fig. 3D). We also detected the SNO-protein level in *Gsnor* TG mice. Results demonstrated that the levels of SNO-protein (Fig. 3E) and *S*-nitrosated PKCα (Fig. 3F) were markedly decreased in the prefrontal cortex tissues of *Gsnor* TG mice compared to WT mice. Moreover, morphine showed minimal effects on SNO-protein and *S*-nitrosated PKCα levels in the prefrontal cortex of *Gsnor* TG mice (Fig. 3*E*–F). Similarly, chronic morphine administration had no effect on PKCα kinase activity in the prefrontal cortex of *Gsnor* TG mice (Fig. 3G). These results suggest that morphine and GSNOR regulate PKCα *S*-nitrosation levels in mouse brain tissues.

### 3.4 PKCα was *S*-nitrosated at Cys78 and Cys132 b y morphine and GSNOR

**Fig. 4:**
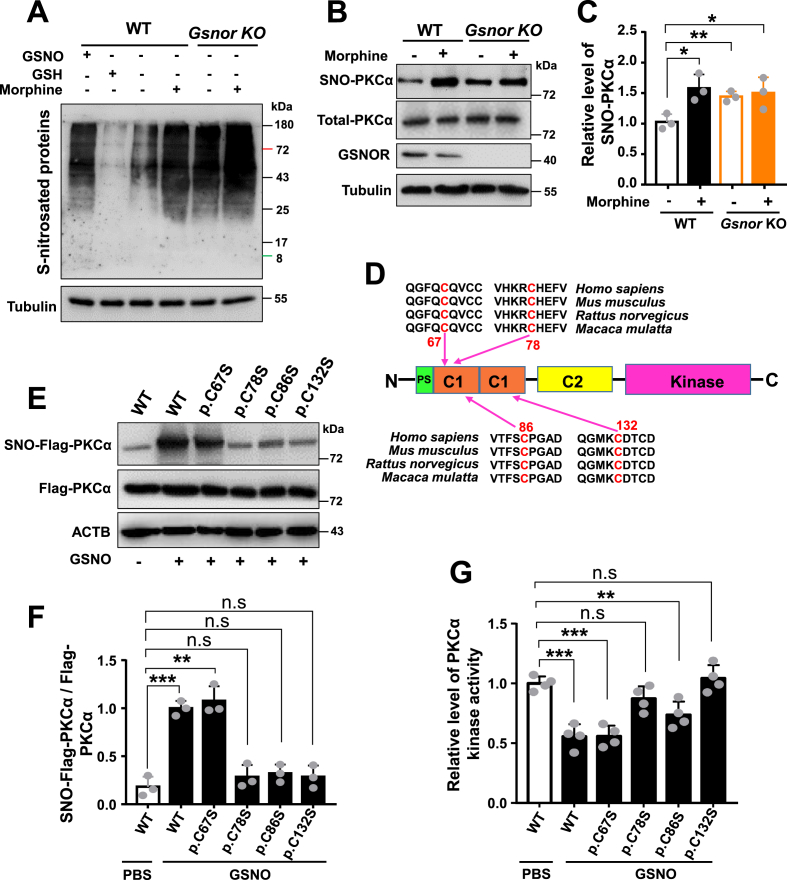
*S*-nitrosation PKCα at Cys78 and Cys132 inhibited PKCα kinase activity in PC12 cells **(A**–**B)** Morphine treatment or *Gsnor* KO increased total protein *S*-nitrosation (**A**) and *S*-nitrosated PKCα levels (**B**) in PC12 cells. Wild-type (WT) and *Gsnor* KO PC12 cells were treated with or without morphine (200 μM) for 24 h before harvest for the subsequent analyses. PC12 cells lysates were incubated with or without GSNO (500 μM) or glutathione (GSH) (500 μM) at room temperature for 30 min, then the protein samples were first reacted with MMTS to block free sulfhydryls. The *S*-nitrosocysteines were then selectively reduced with ascorbate (10 mM) before labeling with the iodoTMTzero reagent. The *anti*-TMT antibody was used for Western blot detection of the TMT-labeled proteins. **(C)** Quantification of PKCα *S*-nitrosation level in (**B**). (**D)** Diagram of mouse PKCα domain structure and PKCα *S*-nitrosation sites Cys67, Cys78, Cys86, and Cys132 identified using the LC-MS/MS analysis. Evolutionary conservation of the predicted sites was evaluated by comparing PKCα protein sequences of humans, macaques, rats, and mice. (**E)** Levels of *S*-nitrosated exogenous PKCα and total exogenous PKCα in PC12 cell lysates after GSNO treatment. Cells were grown in 6-well plate to approach 80 % of confluence, then were transfected with expression vectors (each 2.5 μg) of WT PKCα and cysteine mutants of PKCα (p.C67S, p. C78S, p. C86S, and p. C132S) for 48 h. Cell lysates were collected for biotin-switch assay and PKCα kinase activity detection. Cell lysates were incubated with or without GSNO (500 μM) for 30 min before the biotin-switch assay. (**F**) Quantification of exogenous PKCα *S*-nitrosation level in PC12 cells in (**E**). **(G)** Measurement of relative levels of PKCα kinase activity in WT PKCα and its cysteine mutants (p.C67S, p. C78S, p. C86S, and p. C132S). PC12 cells were transfected and treated with the same procedure in (**E**). To obtain the specific PKCα isoform, immunoprecipitation (IP) was performed before the activity assay. Anti-Flag antibodies were incubated with protein G-agarose beads to form a complex for 1 h at room temperature, followed by the addition of 1 mg of protein and overnight incubation at 4 °C. The beads were collected and washed five times with phosphate-buffered saline (PBS). After sample preparation, PKCα kinase activity was detected according to the manufacturer's protocols of PKC Kinase Activity Assay Kit. All results are representative of three independent experiments with similar results. Values are presented as mean ± SD in all bar graphs. Group differences in (**C**, **F** and **G**) were analyzed by one-way ANOVA with Tukey's *post-hoc* test. *, *P* < 0.05; **, *P* < 0.01; ***, *P* < 0.001; n. s, not significant.

To determine the potential *S*-nitrosation sites of PKCα, we analyzed quantitative proteomics data and identified four cysteine residues (Cys132, Cys86, Cys67, and Cys78) as potential *S*-nitrosation sites (Fig. 3H). We also detected the SNO-protein level in PC12 cells with and without *Gsnor* KO. The SNO-protein levels were substantially increased in cells treated with morphine or subjected to *Gsnor* KO compared to untreated cells (Fig. 4A). Consistent with the *in vivo* results, the *S*-nitrosation levels of PKCα were significantly up-regulated in PC12 WT cells following both morphine treatment and *Gsnor* KO (Fig. 4B–C).

The four potential *S*-nitrosation sites of PKCα (Cys132, Cys86, Cys67, and Cys78) identified by LC-MS/MS analysis were evolutionarily conserved (Fig. 4D). To determine which of these sites played a role in mediating morphine analgesic tolerance, we constructed PKCα expression vectors with individual cysteine mutations to serine. PC12 cells were transfected with expression vectors of WT PKCα containing Flag-tag (Flag-PKCα-WT) and PKCα mutants (p.C67S, p. C78S, p. C86S, and p. C132S), respectively. Cell lysates were harvested and incubated with GSNO for 30 min before the biotin-switch assay. Interestingly, GSNO treatment significantly increased the *S*-nitrosation level of Flag-PKCα-WT and p. C67S of PKCα, while the other PKCα mutants (p.C78S, p. C86S, and p. C132S) partially abolished the *S*-nitrosation of PKCα (Fig. 4*E*–F), suggesting that these three cysteine residues are the major sites of PKCα *S*-nitrosation. Concordantly, the PKCα mutants p. C78S and p. C132S displayed increased PKCα kinase activity when tested using cell lysates of PC12 cells overexpressing these mutants (Fig. 4G). In contrast, the p. C67S and p. C86S mutants did not affect PKCα kinase activity, presenting a similar level of activity as those overexpressing WT PKCα (Fig. 4G). These results indicate that the 78th and 132nd cysteine residues of PKCα serve as major sites regulating PKCα kinase activity through *S*-nitrosation. We also found that inhibition of SNO-PKCα promoted phosphorylation of PKCα at Ser657 (Fig. S7), previously reported to inhibit the kinase activity of PKCα when the serine at Ser657 is changed to alanine [57].

### 3.5 Inhibition of PKCα by GO6976 promoted morphine analgesic tolerance in mice

**Fig. 5:**
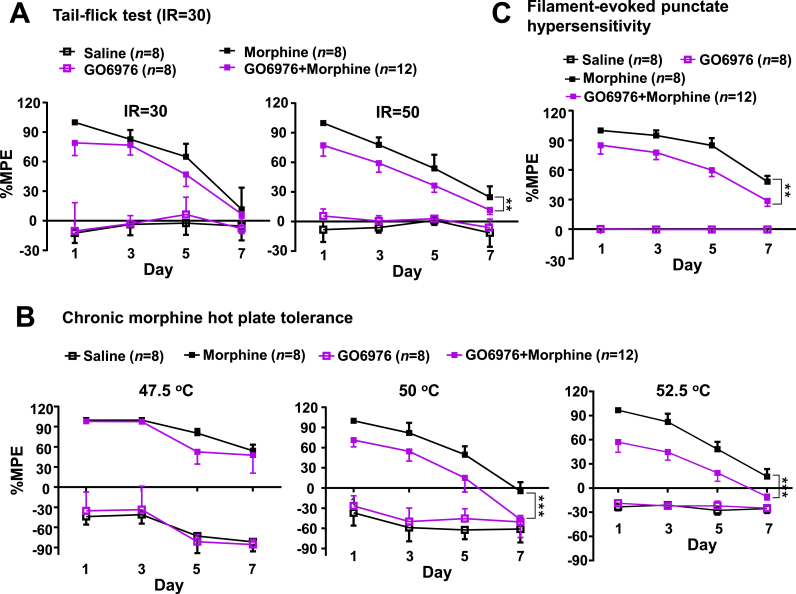
Chemical inhibition of PKCα kinase activity promoted the development of morphine analgesic tolerance in wild-type mice **(A**–**C)** Inhibition of PKCα kinase activity by GO6976 pretreatment before morphine administration in WT mice promoted morphine analgesic tolerance in the tail-flick test (laser density of IR50) (**A**), hot plate tests (50 °C and 52.5 °C) (**B**), and Von Frey test (**C**). WT mice were subjected to behavioral tests on Day 0 (baseline), then received GO6976 (5 mg/kg) or vehicle (DMSO) for 30 min before daily morphine or saline treatment for 7 consecutive days. All results are presented as mean ± SEM. Group differences were analyzed by one-way repeated-measures ANOVA. **, *P* < 0.01; ***, *P* < 0.001.

To further confirm the speculated signaling of morphine-GSNOR-*S*-nitrosated PKCα-reduced PKCα kinase activity during morphine-induced analgesic tolerance, we tested whether treatment by GO6976, a potent and selective inhibitor of PKCα (IC50 value 2.3 nM) and PKCβ1 (IC50 value 6.2 nM) [58,59], would promote the effects of chronic morphine treatment *in vivo*. Mice were subjected to behavioral tests on Day 0 (baseline), then received GO6976 or dimethyl sulfoxide (DMSO) treatment 30 min before daily morphine or saline administration for 7 consecutive days. Pretreatment with GO6976 decreased PKCα kinase activity in the prefrontal cortex tissues of mice (Fig. S8). No differences were observed between the saline and GO6976 groups. However, mice pretreated with GO6976 before morphine injection (GO6976+morphine group) exhibited enhanced morphine analgesic tolerance in the tail-flick test (laser density of IR50) (Fig. 5A), hot plate test (50 °C and 52.5 °C) (Fig. 5B), and Von Frey test (Fig. 5C) compared to animals with morphine treatment only. These results indicated that inhibition of PKCα activity facilitates the development of morphine-induced analgesic tolerance, further confirming that GSNOR-mediated *S*-nitrosation of PKCα is a critical regulator in this process.

## 4 Discussion

**Fig. 6:**
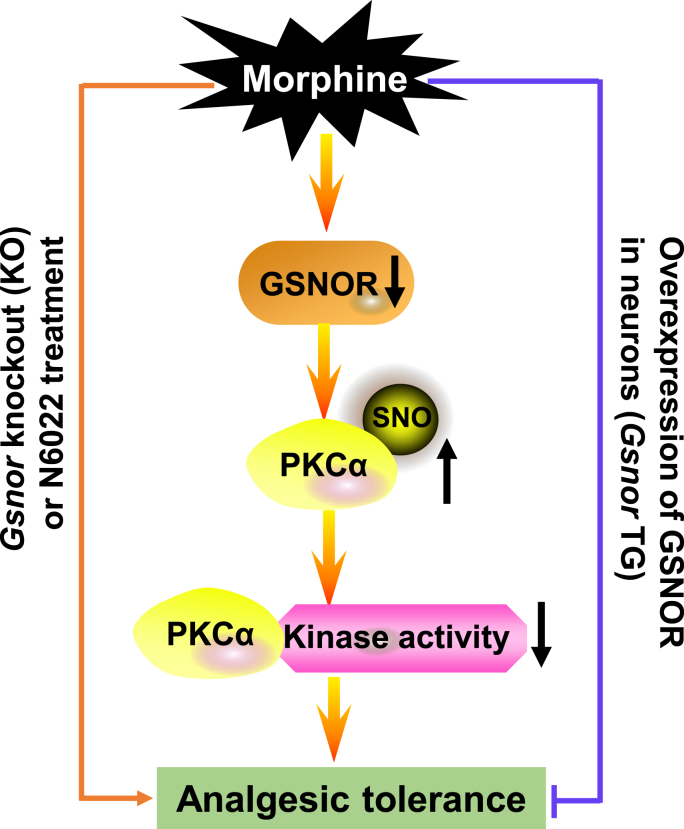
Schematic of potential mechanism underlying the effects of GSNOR on morphine analgesic tolerance Morphine-induced reduction of GSNOR facilitated morphine analgesic tolerance by restricting PKCα cysteine *S*-nitrosation at Cys78 and Cys132, leading to reduced PKCα kinase activity. Knockout or chemical inhibition of GSNOR enhanced this process, while overexpression of *Gsnor* blocked morphine-induced analgesic tolerance.

Morphine, a commonly used pain-relieving medication, is associated with analgesic tolerance and hyperalgesia under prolonged and repeated use [60,61]. The mechanisms underlying the development of morphine tolerance are diverse and complex, with the involvement of multiple signaling pathways [1,62,63]. There were many studies that reported neuronal cell death and apoptosis are involved in morphine analgesic tolerance [47,48]. Excessive production of NO induced by morphine has been implicated in morphine analgesic tolerance [3,64]. Similarly, pretreatment with NOS inhibitors or downregulation of nNOS-1 can block the development of morphine tolerance [[65], [66], [67], [68], [69]]. All these studies indicated that protein *S*-nitrosation, a PTM involved in the attachment of a NO group to a cysteine residue, which has emerged as an important mechanism for post-translational regulation of most or all main classes of proteins and conveys a large part of the ubiquitous influence of NO on cellular signal transduction [21], might be actively involved in morphine addiction and analgesic tolerance. *S-*nitrosation is regulated by the cellular denitrosylase GSNOR [22]. Accumulating evidence has highlighted the importance of GSNOR as a regulator of human health and disease [70]. In the current study, we found that chronic morphine administration led to a reduction in GSNOR expression and an increase in SNO-protein levels in both cellular and mouse models. Thus, we hypothesized that GSNOR-mediated effects may contribute to the development of morphine analgesic tolerance. We obtained several lines of evidence to support this newly proposed mechanism. Firstly, GSNOR deficiency or GSNOR inhibition by N6022 facilitated the development of morphine analgesic tolerance, while overexpression of GSNOR in neurons alleviated this effect. Secondly, the morphine-induced effects were most likely mediated by GSNOR-PKCα signaling, as *Gsnor* KO enhanced PKCα *S*-nitrosation and inhibited its kinase activity, and direct chemical inhibition of PKC activity by GO6976 caused similar behavioral changes as GSNOR deficiency in mouse models. Thirdly, cysteine sites in PKCα that impaired its kinase activity via *S*-nitrosation were identified. These findings provide evidence for a novel signaling pathway involving morphine-GSNOR-*S*-nitrosated PKCα-reduced PKCα kinase activity in the development of morphine analgesic tolerance (Fig. 6), thus offering a potential novel target for modifying *S*-nitrosation in the development of analgesic drugs.

The identification of PKCα as one of the top proteins showing increased SNO-protein levels upon morphine treatment in our proteomics screening assay was not unexpected. Previous studies have implicated PKCα activity in the development of morphine tolerance and dependence [2,71]. Notably, PKCα expression is significantly decreased in the frontal cortex of heroin addicts compared to matched controls [72] and in the frontal cortex of rats after chronic morphine treatment [72,73]. Furthermore, deletion of PKCα also enhances neuropathic mechanical allodynia induced by spared nerve injury [74]. Here, we found that GSNOR mediated changes in the *S*-nitrosation of PKCα and inhibited PKCα kinase activity [54,56], thereby broadening our understanding of the regulatory role of PKCα at the post-translational level in modulating morphine analgesic tolerance. We showed that chronic morphine administration led to *S*-nitrosation of PKCα at the Cys78 and Cys132 residues in the frontal cortex of mice, resulting in decreased kinase activity. Therefore, small molecules that reduce SNO-protein levels, stimulate GSNOR enzymatic activity, or activate PKCα kinase activity, may be promising drug candidates in counteracting morphine-induced analgesic tolerance.

This study has several limitations. Firstly, although we proposed that GSNOR alleviates morphine analgesic tolerance by restricting PKCα *S*-nitrosation at Cys78 and Cys132, this observation should be validated by creating a PKCα^C78S^ and PKCα^C132S^ knock-in mouse model. Furthermore, in our investigation, we employed GO6976 to mimic the inhibition of PKC signaling. This potent but non-selective inhibitor of PKCα could potentially induce non-specific effects, which may somewhat attenuate the robustness of our conclusions. Indeed, previous research has reported that intracerebroventricular injection of PKC inhibitors can prevent the development of antinociceptive tolerance [75], while our study showed that pretreatment with PKCα inhibitors promoted the development of morphine-induced analgesic tolerance. Secondly, we found that GSNOR deficiency induced the *S*-nitrosation of numerous targets based on proteomics analysis of SNO-proteins (Fig. 3B) - this observation is compatible with the involvement of multiple signaling pathways in morphine analgesic tolerance. However, we did not test whether *S*-nitrosation of other targets, in addition to PKCα, plays a key role in affiliating the effects of GSNOR in morphine analgesic tolerance. Examining these prominent candidates, as identified in the proteomic quantification assays, and elucidating the potential neural circuits underpinning this process [76,77] could provide valuable insights. Thirdly, we did not analyze other opioids, such as oxycodone, which exhibits strong morphine-like, pain-relieving effects [78]. Assessing whether GSNOR-mediated *S*-nitrosation of PKCα plays a common role in opioid tolerance warrants further exploration. Finally, we must confess that the proposed GSNOR-mediated *S*-nitrosation of PKCα signaling in morphine analgesic tolerance still needs improvement, and more efforts should be paid to refine the mechanistic clarity. It is ideal to validate the results using non-human primate models before moving forward for potential clinical applications [79].

In summary, we found that repeated morphine administration led to the down-regulation in GSNOR, leading to excessive SNO-protein levels in the prefrontal cortex of mice. Both KO and chemical inhibition of GSNOR promoted the development of morphine analgesic tolerance by affecting the *S*-nitrosation of PKCα and its kinase activity. These findings reveal a novel role of GSNOR in the regulation of morphine analgesic tolerance, highlighting its potential as a therapeutic target for mitigating morphine analgesic tolerance in clinical settings.

## CRediT authorship contribution statement

**Ling-Yan Su:** Conceptualization, Data curation, Formal analysis, Funding acquisition, Investigation, Writing – original draft, Methodology, Project administration, Software. **Lijin Jiao:** Data curation, Investigation, Methodology. **Qianjin Liu:** Data curation, Investigation, Methodology, Funding acquisition. **Xinhua Qiao:** Investigation, Methodology, Funding acquisition. **Ting Xie:** Investigation. **Zhiyu Ma:** Investigation. **Min Xu:** Data curation. **Mao-Sen Ye:** Data curation. **Lu-Xiu Yang:** Methodology. **Chang Chen:** Funding, Supervision, Conceptualization. **Yong-Gang Yao:** Writing – review & editing, Funding acquisition, Supervision, Conceptualization.

## Declaration of competing interest

There were no potential conflicts of interest to be disclosed.
