# Supplementary material for: S-nitrosoglutathione reductase alleviates morphine analgesic tolerance by restricting PKCα S-nitrosation
Source: Redox Biol. 2024 Jun 14;75:103239. doi: 10.1016/j.redox.2024.103239 (PMC11253161; doi:10.1016/j.redox.2024.103239)
Supplement: Multimedia component 1 [file mmc1.docx]

**Online Methods and Data Supplements**

**Cell cultures**

Human neuroblastoma SH-SY5Y cells were obtained from the Kunming Cell Bank, Kunming Institute of Zoology (China). Cells were maintained in Dulbecco’s Modified Eagle Medium (DMEM) (Gibco-BRL, 11965-092) supplemented with 10% fetal bovine serum (FBS, Gibco-BRL, 10099-141), non-essential amino acid (NEEA, Gibco, 11140050), 1 mM sodium pyruvate (Gibco, 11360070), and 1×penicillin/streptomycin (Gibco, 15140122) at 37 °C in a humidified atmosphere incubator with 5% CO_2_ and 95% humidity. Drugs were applied directly to the culture medium for treatment. All experiments were repeated at least three times.

**Cell viability assay**

PC12 and SH-SY5Y cells (1 × 10^4^ cells/well) were seeded in a 96-well plate (100 μL/well). Cells were treated with or without morphine (0 μM, 50 μM, 100 μM, 200 μM) for 24 h. Next, 10 μL of Cell Counting Kit-8 assay (CCK-8) solution (Beyotime, Jiangsu, China) was added to each well at the indicated time. After incubation for 1 h at 37°C, absorbance at 450 nm was measured using an automatic microplate reader (Biotek, Vermont, USA).

**Immunofluorescence**

For immunofluorescence assay, mice were anesthetized with pentobarbital immediately at the end of the cycle of morphine treatment, and were intracardially perfused with saline, followed by 4% paraformaldehyde. The prefrontal cortex was sectioned coronally at 10-μm thickness on a cryostat (Leica, CM1850UV-1-1, Amtzell, Germany). Sections were collected on slides and were incubated with antibodies for neurons (anti-rabbit NeuN, 1: 300, Servicebio, GB11138) and astrocytes (anti-mouse glial fibrillary acidic protein [GFAP], 1:500, Servicebio, GB12096) overnight at 4°C in blocking solution, respectively. After 3 washes (each 5 min) with PBST (phosphate buffered saline [Beyotime Institute of Biotechnology, C0221A] containing Triton X-100 [0.1%; Sigma-Aldrich, 10789704001]), sections were then incubated with a CY3-conjugated anti-Rabbit IgG (1:300; Servicebio, GB21303) or FITC-conjugated anti-mouse IgG (1:400; Servicebio, GB25301) secondary antibody, and nuclei were counterstained with DAPI. The slides were visualized under an Olympus FluoView™ 1000 confocal microscope (Olympus, America).

**Reduced glutathione (GSH) and oxidized glutathione (GSSG) measurement**

The GSH and GSSG content of PC12 cells was determined by a GSH and GSSG Assay Kit (Beyotime, Jiangsu, China) according to the manufacturer's protocol. Briefly, PC12 cells were homogenized in protein-removing reagent M included in the kit at the ratio of 10 mg/30 μL. Then, samples were subjected to two rapid freeze-thaws using liquid nitrogen and a water bath at 37°C, and were left in an ice bath for 5 min. The homogenate was centrifuged at the speed of 10,000 g at 4°C for 10 min. Supernatant was collected and incubated with GSH-masking reagents at room temperature for 1 h. Total glutathione assay working solution were added in the reaction system and kept at room temperature for 5 min, then the chromogenic reaction was started by adding NADPH. Twenty minutes later, absorbance was analyzed using a microplate reader (ELX808; BioTek Instruments, Inc) at 412 nm. The GSSG level was quantified by the standard curve and divided by protein concentration. The GSSG/GSH ratio was calculated as an indicative of oxidative stress.

**Assay for nitric oxide (NO)**

The NO level of the PC12 cells was detected by a total nitric oxide assay kit (Beyotime Institute of Biotechnology, S0023) according to the manufacturer’s instructions. Briefly, PC12 cells were lysed in protein lysis buffer (Beyotime Institute of Biotechnology, P0013) and supernatant was collected by centrifugation at 12000 g for 10 min at 4°C. We added 50 μL supernatant to the Griess reagent and incubated for 10 min, then the nitrite concentration was determined by a microplate reader (ELX808; BioTek Instruments, Inc) at 540 nm.

**DNA extraction and genotype identification**

Mouse genomic DNA was extracted using an AxyPrep™ Multisource Genomic DNA Miniprep Kit (Axygen, AP-MN-MS-GDNA-50). Genotyping was performed using polymerase chain reaction (PCR). Primer pairs are listed in Supplementary Table S2.

**Supplementary Tables**

**Supplementary Table S1.** **Antibodies and chemicals used in this study**

| **Antibodies** | **Source** | **Catalog #** | **WB / IF** |
| --- | --- | --- | --- |
| ***Primary antibody*** |  |  |  |
| Rabbit polyclonal anti-GSNOR | Abcam | ab175406 | 1:1 000 |
| Anti-PKC alpha antibody | Abcam | ab32376 | 1:1 000 |
| Rabbit polyclonal antibody to Phospho-PKC alpha (Ser657) | Affinity | AF8396 | 1:1 000 |
| Mouse monoclonal anti-TMT antibody | ThermoFisher | 90075 | 1:1 000 |
| Mouse monoclonal anti-Flag | Abmart | TT0003 | 1:1 000 |
| Rabbit polyclonal anti-NeuN | Servicebio | GB11138 | 1:300 |
| Mouse monoclonal anti-GFAP | Servicebio | GB12096 | 1:500 |
| Mouse monoclonal anti-tubulin | EnoGene | E1C601 | 1:10 000 |
| Mouse monoclonal anti-ACTB | Beijing Zhong Shan-Golden Bridge Biological Technology CO., LTD | TA-09 | 1:10 000 |
| ***Secondary antibody*** |  |  |  |
| Peroxidase-conjugated anti-rabbit antibody | KPL | 474-1516 | 1:10 000 |
| Peroxidase-conjugated anti-mouse antibody | KPL | 474-1806 | 1:10 000 |
| ***Chemicals*** |  |  |  |
| Morphine hydrochloride | Shenyang Pharmaceutical Co. Ltd. | M0896 | - |
| *S*-nitrosoglutathione (GSNO) | Santa Cruz | sc-200349A | - |
| Glutathione (GSH) | Selleckchem | S4606 | - |
| Ascorbic acid | Selleckchem | S4245 | - |
| Methyl methanethiosulfonate | Sigma-Aldrich | 208795-1G | - |
| Biotin-HPDP | Glpbio | GC11037-100 | - |
| streptavidin-agarose beads | Sigma | S1638 | - |
| GO6976 | Selleckchem | S7119 | - |
| N6022 | TargetMol | T6901 | - |
| Pentobarbital | Sigma | P3761 | - |
| Dimethylsulfoxide (DMSO) | Beyotime | ST038 | - |

WB / IF, Western blot / Immunofluorescence assay

**Supplementary Table S2. Primers for genotyping *Gsnor* KO and *Gsnor* TG mice, as provided by the Jackson laboratory**

| **Primer** | **Sequence (5’-3’)** | **Product length** | **Target** |
| --- | --- | --- | --- |
| *Gsnor KO-1* | TCTTGACGAGTTCTTCTGAGG | 800 bp | *Gsnor* KO |
| *Gsnor KO-2* | CTGAAGCAGCTACTCCCACTAC |  |  |
| *Gsnor WT-1* | TGCCTTCTCGGCTGTGGT | 189 bp | Wild-type |
| *Gsnor WT-2* | GGCCTTTGCGAATTTATCTTTA |  |  |
| *Thy-Gsnor-1* | AGCTTTCCCCACCACAGAATCCAA | 725 bp | *Gsnor* TG |
| *Thy-Gsnor-2* | GGCAGTGTTCACAGCAGCACCATAA |  |  |

**Supplementary Figures**

**Supplementary Figure S1. Measurement of total protein *S*-nitrosation and GSNOR protein level in cultured SH-SY5Y cells**

The SH-SY5Y cells were cultured in DMEM supplemented with 10% FBS, 1×NEEA, 1 mM sodium pyruvate, and 1×penicillin/streptomycin to 80% of confluence, and received morphine treatment (200 μM) for 24 h before harvest. Morphine treatment increased total protein *S*-nitrosation and decreased GSNOR protein level in SH-SY5Y cell lysates. This experiment was repeated three times with consistent results. The procedure for detecting *S*-nitrosated protein is same to that of Figure 1.

**Supplementary Figure S2. Measurement of cytotoxicity of morphine treatment in PC12 and SH-SY5Y cells**

Cell viability was determined by using Cell Counting Kit-8 (CCK8) assay. After morphine exposure for 24 h with the indicated concentrations, cells were incubated with 10 µL of CCK8 reagent for 1 h. Absorbance values were measured at 450 nm. Morphine treatment had no apparent effect on cell viability of PC12 (**A**) and SH-SY5Y (**B**) cells based on the CCK-8 assay. The results are shown as the mean ± SD (*n* = 8). This experiment was repeated three times with consistent results.

**Supplementary Figure S3. Morphine treatment increased the levels of NO and GSSG/GSH ratio in PC12 cells**

The PC12 cells were cultured in DMEM supplemented with 10% FBS and 1×penicillin/streptomycin to 80% of confluence, and received morphine treatment (200 μM) for 24 h. The NO level of PC12 cells was detected by a total nitric oxide assay kit (**A**). The GSH and GSSG content of PC12 cells was determined by a GSH and GSSG Assay Kit (**B**). The results are shown as the mean ± SD (*n* = 6). This experiment was repeated three times with consistent results. Group differences were analyzed by one-way ANOVA with the Tukey’s post-hoc test. **, *P*<0.01; ***, *P*<0.001; ****, *P*<0.0001.

**Supplementary Figure S4. The effect of morphine treatment on neurons and astrocytes in wild-type (WT) mouse prefrontal cortex tissue.** Double-immunostaining of wild-type mouse brain slices with anti-NeuN (red) and anti-GFAP (green) antibodies to show the effects of morphine treatment for 7 consecutive days. Chronic morphine exposure (10 mg/kg; subcutaneous injection for 7 consecutive days) increased the expression of GFAP but not NeuN in the prefrontal cortex tissue of WT mice. DAPI (blue) was used for nuclear staining. Representative images are shown for mice with morphine or saline injection.

**Supplementary Figure S5. Chemical inhibition of GSNOR by N6022 promoted development of morphine analgesic tolerance in WT mice**

Animals were subjected to behavioral tests on Day 0 (baseline), then received N6022 (5 mg/kg) or vehicle (DMSO) for 30 min before daily morphine or saline delivery for 7 consecutive days. Pretreatment with N6022 before morphine injection in WT mice had no effect on the tail-flick test (**A**) but promoted the development of morphine analgesic tolerance in the hot plate (**B**) and Von Frey tests (**C**).

**Supplementary Figure S6. Total protein *S*-nitrosation and *S*-nitrosation of PKCα in prefrontal cortex tissue lysates of WT mice**

(**A**) GSNO treatment increased total protein *S*-nitrosation in prefrontal cortex tissues of WT mice. Mouse prefrontal cortex tissue lysates were incubated with GSNO (500 μM) or glutathione (GSH) (500 μM) at room temperature for 30 min, then the protein samples were first reacted with MMTS to block free sulfhydryls. The *S*-nitrosocysteines were then selectively reduced with ascorbate (10 mM) before labeling with the iodoTMTzero reagent. The anti-TMT antibody was used for Western blot detection of the TMT-labeled proteins.

(**B**) Chronic morphine exposure or pretreatment with N6022 increased *S*-nitrosation level of total protein in prefrontal cortex tissues of WT mice. The procedure for detecting *S*-nitrosated protein is same to that of (**A)**.

(**C**) GSNO treatment increased PKCα *S*-nitrosation in prefrontal cortex tissues of WT mice. Biotinylated proteins of WT mouse prefrontal cortex tissues were immunoprecipitated (IP) with streptavidin-agarose beads, followed by Western blot analysis for PKCα. Tubulin was used as an inner control for protein loading.

**Supplementary Figure S7. Levels of phosphorylation of PKCα at Ser657 in PC12 cells after GSNO treatment**

The PC12 cells were transfected with expression vectors of WT PKCα and its cysteine mutants (p.C67S, p.C78S, p.C86S, and p.C132S) for 48 h, respectively. Cells were incubated with GSNO (50 μM) for 24 h before harvest. Cell lysates were collected to detect phosphorylation of PKCα at Ser657 using Western blot assay. Total PKCα and ACTB were detected as controls.

**Supplementary Figure S8. GO6976 had an inhibition effect on PKCα kinase activity in the prefrontal cortex tissues of WT mice**

WT mice with or without morphine (10 mg/kg), G06976 (5 mg/kg) or a combined administration of both drugs for 7 consecutive days were used for the analysis (n=6 mice per group). These mice were used for the behavioral tests in **Figure 5** before euthanasia for collecting the prefrontal cortex tissues, which were used for quantifying the PKCα kinase activity using the Kinase Activity Assay Kit. Pretreatment of GO6976 inhibited PKCα kinase activity in the cortex tissues of mice (n=6 mice per group) relative to animals without any treatment.
